# Supplementary material for: Cigarette taxation and neonatal and infant mortality: A longitudinal analysis of 159 countries
Source: PLOS Glob Public Health. 2022 Mar 16;2(3):e0000042. doi: 10.1371/journal.pgph.0000042 (PMC10021450; doi:10.1371/journal.pgph.0000042)
Supplement: S1 Table — Abbreviations: VAT = value-added tax; GDP = Gross domestic product; PPP = Purchasing power parity. (DOCX) [file pgph.0000042.s001.docx]

S1 Table. Summary statistics of the included variables (2008-2018)

| Variable | Number of observations | Mean | Standard deviation | Interquartile range |
| --- | --- | --- | --- | --- |
| Neonatal mortality (per 1000 live births) | 2134 | 14.4 | 11.7 | 4.4 – 22.9 |
| Infant mortality (per 1000 live births) | 2134 | 24.9 | 22.7 | 6.8 – 38.6 |
| Cigarette consumption per capita | 432 | 1490.3 | 809.9 | 905.5 – 1922.5 |
| Total tax (% of retail price) | 2103 | 49.1 | 22.6 | 30.1 – 71.0 |
| Specific tax (% of retail price) | 2103 | 19.5 | 20.4 | 0 – 34.1 |
| Ad valorem tax (% of retail price) | 2103 | 14.7 | 18.8 | 0 – 25.0 |
| Import duties, VAT, and other taxes (% of retail price) | 2103 | 14.9 | 10.7 | 9.7 – 16.7 |
| Protecting people from tobacco smoke | 2145 | 2.1 | 1.2 | 1 – 3 |
| Offering help to quit tobacco use | 2145 | 2.6 | 0.8 | 2 – 3 |
| Warning about the dangers of tobacco – Health warnings | 2145 | 2.3 | 1.2 | 1 – 3 |
| Warning about the dangers of tobacco – Mass media | 2145 | 1.7 | 1.2 | 1 – 2.5 |
| Enforcing bans on tobacco advertising, promotion and sponsorship | 2145 | 2.5 | 1.1 | 1 – 3 |
| GDP (PPP per 1000) | 2057 | 17.9 | 19.8 | 3.5 – 24.9 |
| Rural population (%) | 2123 | 42.6 | 23.1 | 23.4 – 61.6 |
| Fertility rate | 2048 | 2.9 | 1.4 | 1.8 – 3.9 |
| Access to safe drinking water (%) | 2123 | 86.0 | 17.4 | 76.9 – 99.1 |
| Health expenditure (PPP per 1000) | 2058 | 1.3 | 1.6 | 0.2 – 1.7 |
| Female primary education completion rate (%) | 1862 | 88.7 | 21.4 | 81.6 – 100.6 |
| Clean cooking (%) | 2079 | 63.7 | 37.9 | 24.6 – 98.1 |
| Total primary education completion rate | 1875 | 89.4 | 19.8 | 81.1 – 100.4 |

Abbreviations: VAT= value-added tax; GDP= Gross domestic product; PPP= Purchasing power parity
