# Supplementary material for: Cigarette taxation and neonatal and infant mortality: A longitudinal analysis of 159 countries
Source: PLOS Glob Public Health. 2022 Mar 16;2(3):e0000042. doi: 10.1371/journal.pgph.0000042 (PMC10021450; doi:10.1371/journal.pgph.0000042)
Supplement: S2 Table — Abbreviations: VAT = value-added tax; GDP = Gross domestic product; PPP = Purchasing power parity. (DOCX) [file pgph.0000042.s002.docx]

**S2 Table. Summary statistics of the included variables according to income groups (2008-2018)**

| **Predictor variables** | **Low- and middle-income countries** | | | | **High-income countries** | | | |
| --- | --- | --- | --- | --- | --- | --- | --- | --- |
|  | **Number of observations** | **Mean** | **Standard deviation** | **Interquartile range** | **Number of observations** | **Mean** | **Standard deviation** | **Interquartile range** |
| Neonatal mortality (per 1000 live births) | 1485 | 19.0 | 11.1 | 9.6 – 26.9 | 627 | 3.7 | 2.7 | 2.0 – 4.6 |
| Infant mortality (per 1000 live births) | 1485 | 33.23 | 22.4 | 14.8 – 47.7 | 627 | 5.6 | 4.0 | 3.0 – 6.8 |
| Cigarette consumption per capita (% of retail price ) | 227 | 1470.5 | 998.0 | 573.0 – 2138.0 | 205 | 1512.2 | 530.7 | 1098.0 – 1808.0 |
| Total tax (% of retail price) | 1465 | 42.7 | 20.7 | 27.7 – 56.3 | 616 | 63.7 | 19.9 | 57.1 – 77.3 |
| Specific tax (% of retail price) | 1465 | 15.6 | 18.1 | 0 – 29.3 | 616 | 28.6 | 22.4 | 7.1 – 46.5 |
| Ad valorem tax (% of retail price) | 1465 | 13.0 | 17.6 | 0 – 19.9 | 616 | 19.3 | 20.8 | 0 -– 34.0 |
| Import duties, VAT, and other taxes (% of retail price) | 1465 | 14.1 | 10.0 | 9.1 – 16.0 | 616 | 15.9 | 9.1 | 13.0 – 18.7 |
| Protecting people from tobacco smoke | 1496 | 2.1 | 1.2 | 1 – 3 | 627 | 2.1 | 1.3 | 1 – 3 |
| Offering help to quit tobacco use | 1496 | 2.3 | 0.8 | 2 – 3 | 627 | 3.1 | 0.7 | 3 – 4 |
| Warning about the dangers of tobacco – Health warnings | 1496 | 2.1 | 1.2 | 1 – 3 | 627 | 2.6 | 1.1 | 1 – 4 |
| Warning about the dangers of tobacco – Mass media | 1496 | 1.6 | 1.2 | 1 – 2.5 | 627 | 2.0 | 1.2 | 1 – 3 |
| Enforcing bans on tobacco advertising, promotion and sponsorship | 1496 | 2.5 | 1.1 | 1 – 3 | 627 | 2.6 | 0.9 | 2 – 3 |
| GDP (PPP per 1000) | 1452 | 8.0 | 6.4 | 2.7 – 12.1 | 605 | 41.5 | 20.9 | 27.4 – 48.7 |
| Rural population (%) | 1496 | 50.6 | 20.4 | 34.0 – 66.8 | 627 | 23.5 | 17.1 | 11.8 – 32.6 |
| Fertility rate | 1453 | 80.5 | 18.1 | 66.3 – 95.7 | 595 | 1.8 | 0.4 | 1.5 – 2.0 |
| Access to safe drinking water (%) | 1496 | 59.8 | 25.9 | 42.2 – 81.9 | 627 | 99.0 | 2.0 | 98.9 – 100.0 |
| Health expenditure (PPP per 1000) | 1431 | 0.5 | 0.4 | 0.1 – 0.7 | 627 | 3.1 | 1.7 | 1.8 – 4.2 |
| Female primary education completion rate | 1366 | 84.8 | 22.7 | 71.6 – 99.5 | 496 | 99.5 | 11.9 | 95.9 – 102.6 |
| Clean cooking (%) | 1452 | 48.8 | 36.3 | 11.3 – 87.3 | 627 | 98.1 | 3.5 | 97.5 – 100.0 |
| Total primary education completion rate | 1357 | 85.7 | 21.5 | 72.3 – 143.6 | 518 | 99.0 | 8.8 | 95.9 – 102.0 |

Abbreviations: VAT= value-added tax; GDP= Gross domestic product; PPP= Purchasing power parity
