# Supplementary material for: Cigarette taxation and neonatal and infant mortality: A longitudinal analysis of 159 countries
Source: PLOS Glob Public Health. 2022 Mar 16;2(3):e0000042. doi: 10.1371/journal.pgph.0000042 (PMC10021450; doi:10.1371/journal.pgph.0000042)
Supplement: S3 Table — Note: We reported ratios (i.e. exponential values of effect estimates) from regression models with log-transformed neonatal and infant mortality outcomes. Hausman Test indicated for each model that fixed effect model is the preferred model. Abbreviations: VAT = value-added tax; GDP = Gross domestic product; PPP = Purchasing power parity, AIC = Akaike information criterion; BIC = Bayesian information criterion. (DOCX) [file pgph.0000042.s003.docx]

**S3 Table. Results from the fixed effects panel regression model for the association between total taxes and neonatal and infant mortality (Ratios and 95% Confidence Interval)**

| **Predictor variables** | **Neonatal mortality** | | | | **Infant mortality** | | | |
| --- | --- | --- | --- | --- | --- | --- | --- | --- |
|  | **Overall - tax as continuous variable** | **Overall - tax in quartiles** | **High-income countries** | **Low- and middle-income countries** | **Overall - tax as continuous variable** | **Overall - tax in quartiles** | **High-income countries** | **Low- and middle-income countries** |
| **Total tax (per 10%)** | 0.974  (0.968; 0.981) | – | 0.986  (0.973; 1.001) | 0.974  (0.967; 0.981) | 0.981  (0.974; 0.987) | – | 0.993  (0.979; 1.007) | 0.980  (0.973; 0.987) |
| Total tax: 0%-24.9% | – | (R) | – | – | – | (R) | – | – |
| Total tax: 25%-44.9% | – | 0.967  (0.946; 0.988) | – | – | – | 0.974  (0.953; 0.996) | – | – |
| Total tax: 45%-74.9% | – | 0.929  (0.903; 0.956) | – | – | – | 0.948  (0.921; 0.974) | – | – |
| Total tax: 75%-max | – | 0.896  (0.864; 0.928) | – | – | – | 0.911  (0.879; 0.944) | – | – |
| Protecting people from tobacco smoke | 0.993  (0.987; 1.000) | 0.994  (0.987; 1.001) | 0.993  (0.980; 1.007) | 1.000  (0.993; 1.006) | 0.994  (0.987; 1.000) | 0.994  (0.988; 1.001) | 0.993  (0.979; 1.007) | 0.998  (0.991; 1.005) |
| Offering help to quit tobacco use | 0.993  (0.982; 1.003) | 0.994  (0.984; 1.005) | 0.989  (0.966; 1.013) | 1.000  (0.990; 1.011) | 0.986  (0.976; 0.997) | 0.987  (0.977; 0.998) | 0.997  (0.974; 1.020) | 0.989  (0.978; 0.999) |
| Warning about the dangers of tobacco – Health warnings | 0.978  (0.971; 0.986) | 0.977  (0.970; 0.985) | 0.960  (0.946; 0.974) | 0.989  (0.982; 0.997) | 0.973  (0.966; 0.981) | 0.973  (0.966; 0.980) | 0.952  (0.939; 0.966) | 0.984  (0.976; 0.992) |
| Warning about the dangers of tobacco – Mass media | 1.000  (0.996; 1.005) | 1.000  (0.996; 1.005) | 0.991  (0.982; 0.999) | 1.005  (1.000; 1.009) | 1.005  (1.000; 1.009) | 1.004  (1.000; 1.009) | 0.994  (0.985; 1.002) | 1.009  (1.005; 1.014) |
| Enforcing bans on TAPS | 0.998  (0.990; 1.006) | 0.997  (0.989; 1.005) | 0.998  (0.978; 1.019) | 0.994  (0.986; 1.002) | 0.996  (0.987; 1.004) | 0.995  (0.987; 1.003) | 0.973  (0.954; 0.993) | 0.998  (0.99; 1.006) |
| GDP (PPP per 1000) | 0.997  (0.996; 0.998) | 0.997  (0.996; 0.999) | 0.999  (0.997; 1.000) | 0.991  (0.988; 0.994) | 0.997  (0.996; 0.998) | 0.997  (0.996; 0.998) | 0.998  (0.997; 1.000) | 0.990  (0.986; 0.993) |
| Rural population (per 10%) | 1.121  (1.079; 1.165) | 1.131  (1.087; 1.175) | 0.725  (0.636; 0.827) | 1.123  (1.083; 1.164) | 1.139  (1.096; 1.184) | 1.146  (1.102; 1.190) | 0.716  (0.629; 0.815) | 1.139  (1.098; 1.181) |
| Fertility rate | 1.072  (1.040; 1.106) | 1.070  (1.037; 1.104) | 1.232  (1.120; 1.357) | 1.089  (1.057; 1.122) | 1.160  (1.124; 1.196) | 1.157  (1.122; 1.194) | 1.298  (1.181; 1.428) | 1.185  (1.151; 1.220) |
| Drinking water (per 10%) | 0.951  (0.928; 0.974) | 0.951  (0.928; 0.975) | 0.895  (0.780; 1.026) | 0.955  (0.935; 0.976) | 0.945  (0.922; 0.968) | 0.944  (0.922; 0.968) | 0.882  (0.772; 1.010) | 0.950  (0.930; 0.971) |
| Health expenditure (PPP per 1000) | 0.904  (0.890; 0.919) | 0.904  (0.889; 0.919) | 0.940  (0.920; 0.961) | 0.728  (0.698; 0.761) | 0.897  (0.882; 0.911) | 0.897  (0.882; 0.911) | 0.934  (0.914; 0.954) | 0.709  (0.679; 0.741) |
| Female primary education completion rate (per 10) | 1.004  (0.999; 1.009) | 1.005  (1.000; 1.010) | 0.997  (0.988; 1.006) | 0.999  (0.993; 1.005) | 1.003  (0.998; 1.008) | 1.003  (0.998; 1.008) | 0.996  (0.987; 1.005) | 0.999  (0.993; 1.004) |
| Clean cooking (per 10%) | 0.920  (0.902; 0.937) | 0.917  (0.899; 0.934) | 0.516  (0.435; 0.612) | 0.947  (0.931; 0.963) | 0.923  (0.906; 0.941) | 0.921  (0.904; 0.939) | 0.559  (0.473; 0.662) | 0.955  (0.939; 0.972) |
| N (number of observations) | 1709 | 1709 | 475 | 1234 | 1709 | 1709 | 475 | 1234 |
| Hausman test (P-value) | p < 0.001 | p < 0.001 | p < 0.001 | p < 0.001 | p < 0.001 | p < 0.001 | p < 0.001 | p < 0.001 |

Note: We reported ratios (i.e. exponential values of effect estimates) from regression models with log-transformed neonatal and infant mortality outcomes. Hausman Test indicated for each model that fixed effect model is the preferred model.

Abbreviations: VAT= value-added tax; GDP= Gross domestic product; PPP= Purchasing power parity, AIC= Akaike information criterion; BIC= Bayesian information criterion
