# Supplementary material for: Cigarette taxation and neonatal and infant mortality: A longitudinal analysis of 159 countries
Source: PLOS Glob Public Health. 2022 Mar 16;2(3):e0000042. doi: 10.1371/journal.pgph.0000042 (PMC10021450; doi:10.1371/journal.pgph.0000042)
Supplement: S4 Table — Note: We reported ratios (i.e. exponential values of effect estimates) from regression models with log-transformed neonatal and infant mortality outcomes. Hausman Test indicated for each model that fixed effect model is the preferred model. Abbreviations: VAT = value-added tax; GDP = Gross domestic product; PPP = Purchasing power parity, AIC = Akaike information criterion; BIC = Bayesian information criterion. (DOCX) [file pgph.0000042.s004.docx]

**S4 Table. Results from the fixed effects panel regression model for the association between different types of taxes and neonatal and infant mortality (Ratios and 95% Confidence Interval)**

| **Predictor variables** | **Neonatal mortality** | **Infant mortality** | |
| --- | --- | --- | --- |
| **Specific tax (per 10%)** | 0.977  (0.970; 0.984) | | 0.983  (0.976; 0.990) |
| **Ad valorem (per 10%)** | 0.975  (0.967; 0.983) | | 0.983  (0.975; 0.991) |
| **Import duties, VAT, and other taxes (per 10%)** | 0.961  (0.950; 0.972) | | 0.965  (0.954; 0.976) |
| Protecting people from tobacco smoke | 0.993  (0.987; 1.000) | | 0.993  (0.987; 1.000) |
| Offering help to quit tobacco use | 0.993  (0.983; 1.004) | | 0.987  (0.976; 0.997) |
| Warning about the dangers of tobacco – Health warnings | 0.977  (0.970; 0.984) | | 0.972  (0.965; 0.979) |
| Warning about the dangers of tobacco – Mass media | 1.000  (0.996; 1.005) | | 1.004  (1.000; 1.009) |
| Enforcing bans on tobacco advertising, promotion and sponsorship | 0.996  (0.988; 1.005) | | 0.994  (0.985; 1.002) |
| GDP (PPP per 1000) | 0.997  (0.996; 0.999) | | 0.997  (0.996; 0.998) |
| Rural population (per 10%) | 1.122  (1.079; 1.166) | | 1.140  (1.096; 1.184) |
| Fertility rate | 1.073  (1.040; 1.106) | | 1.160  (1.124; 1.196) |
| Drinking water (per 10%) | 0.948  (0.926; 0.972) | | 0.942  (0.920; 0.965) |
| Health expenditure (PPP per 1000) | 0.903  (0.889; 0.918) | | 0.896  (0.882; 0.910) |
| Female primary education completion rate (per 10) | 1.007  (1.001; 1.012) | | 1.006  (1.000; 1.011) |
| Clean cooking (per 10%) | 0.920  (0.903; 0.938) | | 0.925  (0.907; 0.942) |
| N (number of observations) | 1709 | | 1709 |
| Hausman test (P-value) | p < 0.001 | | p < 0.001 |

Note: We reported ratios (i.e. exponential values of effect estimates) from regression models with log-transformed neonatal and infant mortality outcomes. Hausman Test indicated for each model that fixed effect model is the preferred model.

Abbreviations: VAT= value-added tax; GDP= Gross domestic product; PPP= Purchasing power parity, AIC= Akaike information criterion; BIC= Bayesian information criterion
