# Supplementary material for: Cigarette taxation and neonatal and infant mortality: A longitudinal analysis of 159 countries
Source: PLOS Glob Public Health. 2022 Mar 16;2(3):e0000042. doi: 10.1371/journal.pgph.0000042 (PMC10021450; doi:10.1371/journal.pgph.0000042)
Supplement: S5 Table — Note: Hausman Test indicated for each model that fixed effect model is the preferred model, except in case of the model for high-income countries (in this case the preferred model is the random effect model that would give the following B-values [95% Confidence interval]: -119.1 [-198.5; -49.6]). Abbreviations: VAT = value-added tax; GDP = Gross domestic product; PPP = Purchasing power parity, AIC = Akaike information criterion; BIC = Bayesian information criterion. (DOCX) [file pgph.0000042.s005.docx]

**S5 Table. Results from the fixed-effects panel regression model for the association between taxes and cigarette consumption without any control variables (B-value and 95% Confidence Interval)**

| **Predictor variables** | **Overall - tax as continuous variable** | **Overall - tax in quartiles** | **High-income countries** | **Low- and middle-income countries** | **Different types of taxes** |
| --- | --- | --- | --- | --- | --- |
| **Total tax (per 10%)** | -160.9  (-223.8; -97.9) | – | -233.1  (-375.2; -91.03) | -148.8  (-225.2; -72.5) | – |
| **Total tax: 0%-24.9%** | – | (R) | – | – | – |
| **Total tax: 25%-44.9%** | – | -144.1  (-415.3; 127.1) | – | – | – |
| **Total tax: 45%-74.9%** | – | -289.0  (-604.7; 26.71) | – | – | – |
| **Total tax: 75%-max** | – | -684.8  (-1028.8; -340.8) | – | – | – |
| **Specific tax (per 10%)** | – | – | – | – | -150.4  (-214.3; -86.5) |
| **Ad valorem (per 10%)** | – | – | – | – | -86.4  (-154.6; -18.3) |
| **Import duties, VAT, and other taxes (per 10%)** | – | – | – | – | -782.2  (-1045.7; -518.6) |
| N (number of observations) | 432 | 432 | 205 | 227 | 432 |
| Hausman test (P-value) | p < 0.001 | 0.01 | 0.06 | 0.03 | p < 0.001 |

Note: Hausman Test indicated for each model that fixed effect model is the preferred model, except in case of the model for high-income countries (in this case the preferred model is the random effect model that would give the following B-values [95% Confidence interval]: -119.1 [-198.5; -49.6])

Abbreviations: VAT= value-added tax; GDP= Gross domestic product; PPP= Purchasing power parity, AIC= Akaike information criterion; BIC= Bayesian information criterion
