# Supplementary material for: Cigarette taxation and neonatal and infant mortality: A longitudinal analysis of 159 countries
Source: PLOS Glob Public Health. 2022 Mar 16;2(3):e0000042. doi: 10.1371/journal.pgph.0000042 (PMC10021450; doi:10.1371/journal.pgph.0000042)
Supplement: S6 Table — Note: Hausman Test indicated for each model that fixed effect model is the preferred model, except in case of the model for high-income countries (in this case the preferred model is the random effect model that would give the following B-values [95% Confidence interval]: -84.1 [-155.2; -12.9]). Abbreviations: VAT = value-added tax; GDP = Gross domestic product; PPP = Purchasing power parity, AIC = Akaike information criterion; BIC = Bayesian information criterion. (DOCX) [file pgph.0000042.s006.docx]

**S6 Table. Results from the fixed-effects panel regression model for the association between taxes and cigarette consumption (B-value and 95% Confidence Interval)**

| **Predictor variables** | **Overall Total** | **Overall Quartiles** | **High-income countries** | **Low- and middle-income countries** | **Different types of taxes** |
| --- | --- | --- | --- | --- | --- |
| **Total tax (per 10%)** | -94.6  (-156.5; -32.7) | – | -105.5  (-258.8; 47.9) | -86.2  (-167.3; -5.03) | – |
| **Total tax: 0%-24.9%** | – | (R) | – | – | – |
| **Total tax: 25%-44.9%** | – | -15.5  (-283.3; 252.3) | – | – | – |
| **Total tax: 45%-74.9%** | – | -144.6  (-441.5; 152.2) | – | – | – |
| **Total tax: 75%-max** | – | -377.8  (-708.9; -46.6) | – | – | – |
| **Specific tax (per 10%)** | – | – | – | – | -88.5  (-155.9; -21.1) |
| **Ad valorem (per 10%)** | – | – | – | – | -38.3  (-105.1; 28.5) |
| **Import duties, VAT, and other taxes (per 10%)** | – | – | – | – | -678.4  (-970.2; -386.7) |
| Protecting people from tobacco smoke | -82.6  (-135.6; -29.53) | -76.7  (-130.1; -23.3) | -159.6  (-240.7; -78.4) | -49.9  (-125.3; 25.4) | -93.3  (-145.2; -41.3) |
| Offering help to quit tobacco use | -272.7  (-364; -181.4) | -256.8  (-350.3; -163.3) | -305.0  (-447; -163.1) | -253.7  (-384.9; -122.5) | -207.4  (-301.1; -113.7) |
| Warning about the dangers of tobacco; Health warnings | 59.6  (-12.1; 131.3) | 46.4  (-24.9; 117.7) | 89.2  (-29.6; 207.9) | 28.3  (-70.3; 126.8) | 33.7  (-37.8; 105.2) |
| Warning about the dangers of tobacco; Mass media | 78.2  (44.7; 111.6) | 74.1  (40.2; 108.1) | 46.4  (3.4; 89.4) | 89.1  (34.9; 143.4) | 63.3  (29.5; 97.2) |
| Enforcing bans on TAPS | 34.3  (-36.4; 105.0) | 16.6  (-56.7; 89.8) | -23.6  (-154.6; 107.5) | 41.7  (-56.0; 139.3) | 63.2  (-6.7; 133.0) |
| GDP (PPP per 1000) | -24.1  (-38.5; -9.6) | -23.0  (-37.4; -8.6) | -29.9  (-45.1; -14.6) | -18.2  (-56.7; 20.4) | -17.73  (-32.02; -3.432) |
| Fertility rate | -118.3  (-514.1; 277.5) | -141.6  (-553.6; 270.4) | 181.3  (-444.2; 806.9) | -179.6  (-746.3; 387.1) | 34.9  (-376.4; 446.1) |
| Total primary education completion rate (per 10) | -2.8  (-75.1; 69.5) | -5.5  (-77.7; 66.7) | 110.2  (-30.2; 250.5) | -31.4  (-124.1; 61.4) | -28.3  (-100.5; 43.8) |
| N (number of observations) | 355 | 355 | 161 | 194 | 355 |
| Hausman test (P-value) | p < 0.001 | 0.01 | 0.40 | 0.04 | 0.01 |

Note: Hausman Test indicated for each model that fixed effect model is the preferred model, except in case of the model for high-income countries (in this case the preferred model is the random effect model that would give the following B-values [95% Confidence interval]: -84.1 [-155.2; -12.9])

Abbreviations: VAT= value-added tax; GDP= Gross domestic product; PPP= Purchasing power parity, AIC= Akaike information criterion; BIC= Bayesian information criterion
