# Supplementary material for: Cigarette taxation and neonatal and infant mortality: A longitudinal analysis of 159 countries
Source: PLOS Glob Public Health. 2022 Mar 16;2(3):e0000042. doi: 10.1371/journal.pgph.0000042 (PMC10021450; doi:10.1371/journal.pgph.0000042)
Supplement: S7 Table — Note: We reported ratios (i.e. exponential values of effect estimates) from regression models with log-transformed neonatal and infant mortality outcomes. Hausman Test indicated for each model that fixed effect model is the preferred model. Abbreviations: VAT = value-added tax; GDP = Gross domestic product; PPP = Purchasing power parity, AIC = Akaike information criterion; BIC = Bayesian information criterion. (DOCX) [file pgph.0000042.s007.docx]

**S7 Table. Results from the fixed-effects panel regression model for neonatal and infant mortality (Ratios and 95% Confidence Interval) and cigarette consumption (B-value and 95% Confidence Interval) without missing value imputation**

| **Predictor variables** | **Neonatal mortality** | **Infant mortality** | **Cigarette Consumption** |
| --- | --- | --- | --- |
| **Total tax** | 0.969 (0.956; 0.983) | 0.979 (0.965; 0.993) | -249.8 (-362.4; -137.2) |
| Measurement for protecting people from tobacco smoke | 0.993 (0.980; 1.005) | 0.993 (0.981; 1.005) | -91.13 (-181.7; -0.6) |
| Measurement for offering help to quit tobacco use | 0.978 (0.958; 0.998) | 0.975 (0.956; 0.995) | -304.4 (-469.7; -139.2) |
| Warning about the dangers of tobacco – Health warnings | 0.990 (0.977; 1.003) | 0.985 (0.972; 0.997) | 74.2 (-56.9; 205.3) |
| Warning about the dangers of tobacco – Mass media | 1.000 (0.993; 1.007) | 1.003 (0.996; 1.010) | 70.8 (16.0; 125.5) |
| Measurement for enforcing bans on tobacco advertising, promotion and sponsorship | 1.006 (0.991; 1.021) | 1.001 (0.987; 1.016) | 112.0 (-8.2; 232.1) |
| GDP (PPP per 1000) | 0.998 (0.995; 1.000) | 0.997 (0.995; 1.000) | -30.0 (-60.2; 0.1) |
| Fertility rate | 1.066 (0.993; 1.143) | 1.150 (1.073; 1.232) | -377.9 (-1120.5; 364.8) |
| Rural population (per 10%) | 1.042 (0.954; 1.139) | 1.043 (0.956; 1.139) | – |
| Drinking water (per 10%) | 0.889 (0.843; 0.938) | 0.873 (0.828; 0.920) | – |
| Health expenditure (per 1000) | 0.882 (0.853; 0.912) | 0.874 (0.845; 0.903) | – |
| Female primary education completion rate (per 10%) | 0.994 (0.979; 1.009) | 0.989 (0.975; 1.004) | – |
| Clean cooking (per 10%) | 0.890 (0.855; 0.928) | 0.890 (0.856; 0.927) | – |
| Total primary education completion rate (per 10) | – | – | 11.05 (-137.8; 159.9) |
| N (number of observations) | 564 | 564 | 149 |
| Hausman test (P-value) | p < 0.001 | p < 0.001 | 0.01 |

Note: We reported ratios (i.e. exponential values of effect estimates) from regression models with log-transformed neonatal and infant mortality outcomes. Hausman Test indicated for each model that fixed effect model is the preferred model.

Abbreviations: VAT= value-added tax; GDP= Gross domestic product; PPP= Purchasing power parity, AIC= Akaike information criterion; BIC= Bayesian information criterion
