# Supplementary material for: Cigarette taxation and neonatal and infant mortality: A longitudinal analysis of 159 countries
Source: PLOS Glob Public Health. 2022 Mar 16;2(3):e0000042. doi: 10.1371/journal.pgph.0000042 (PMC10021450; doi:10.1371/journal.pgph.0000042)
Supplement: S8 Table — Note: We reported ratios (i.e. exponential values of effect estimates) from regression models with log-transformed neonatal and infant mortality outcomes. Hausman Test indicated for each model that fixed effect model is the preferred model. Abbreviations: VAT = value-added tax; GDP = Gross domestic product; PPP = Purchasing power parity, AIC = Akaike information criterion; BIC = Bayesian information criterion Akaike information criterion; BIC = Bayesian information criterion. (DOCX) [file pgph.0000042.s008.docx]

**S8 Table. Results from the fixed effects panel regression model for the association between total taxes and neonatal and infant mortality excluding education variables (Ratios and 95% Confidence Interval)**

| **Predictor variables** | **Neonatal mortality** | | | | **Infant mortality** | | | |
| --- | --- | --- | --- | --- | --- | --- | --- | --- |
|  | **Overall - tax as continuous variable** | **Overall - tax in quartiles** | **High-income countries** | **Low- and middle-income countries** | **Overall - tax as continuous variable** | **Overall - tax in quartiles** | **High-income countries** | **Low- and middle-income countries** |
| **Total tax (per 10%)** | 0.975  (0.969; 0.982) |  | 0.990  (0.977; 1.003) | 0.974  (0.967; 0.980) | 0.981  (0.975; 0.988) |  | 0.996  (0.983; 1.009) | 0.980  (0.973; 0.986) |
| Total tax: 0%-24.9% |  | (R) |  |  |  | (R) |  |  |
| Total tax: 25%-44.9% |  | 0.969  (0.948; 0.990) |  |  |  | 0.976  (0.956; 0.997) |  |  |
| Total tax: 45%-74.9% |  | 0.931  (0.906; 0.957) |  |  |  | 0.949  (0.924; 0.975) |  |  |
| Total tax: 75%-max |  | 0.898  (0.868; 0.929) |  |  |  | 0.912  (0.882; 0.943) |  |  |
| Protecting people from tobacco smoke | 0.990  (0.984; 0.997) | 0.991  (0.985; 0.997) | 0.995  (0.983; 1.008) | 0.998  (0.991; 1.004) | 0.991  (0.985; 0.997) | 0.991  (0.985; 0.998) | 0.994  (0.982; 1.006) | 0.997  (0.990; 1.003) |
| Offering help to quit tobacco use | 0.989  (0.979; 0.999) | 0.990  (0.98; 1.000) | 0.984  (0.965; 1.003) | 0.997  (0.987; 1.007) | 0.986  (0.977; 0.996) | 0.987  (0.978; 0.997) | 0.999  (0.981; 1.018) | 0.987  (0.977; 0.997) |
| Warning about the dangers of tobacco– Health warnings | 0.981  (0.974; 0.988) | 0.981  (0.974; 0.987) | 0.962  (0.950; 0.974) | 0.992  (0.985; 0.999) | 0.976  (0.969; 0.983) | 0.976  (0.969; 0.982) | 0.956  (0.945; 0.968) | 0.986  (0.979; 0.993) |
| Warning about the dangers of tobacco – Mass media | 1.001  (0.997; 1.005) | 1.001  (0.997; 1.005) | 0.993  (0.985; 1.001) | 1.005  (1.001; 1.009) | 1.005  (1.001; 1.010) | 1.005  (1.001; 1.009) | 0.995  (0.988; 1.003) | 1.010  (1.005; 1.014) |
| Enforcing bans on TAPS | 0.997  (0.989; 1.005) | 0.996  (0.988; 1.004) | 1.004  (0.985; 1.024) | 0.993  (0.985; 1.000) | 0.994  (0.987; 1.002) | 0.994  (0.986; 1.001) | 0.979  (0.962; 0.998) | 0.996  (0.989; 1.004) |
| GDP (PPP per 1000) | 0.998  (0.997; 0.999) | 0.998  (0.997; 0.999) | 0.999  (0.998; 1.000) | 0.991  (0.988; 0.994) | 0.998  (0.997; 0.999) | 0.998  (0.997; 0.999) | 0.999  (0.998; 1.000) | 0.990  (0.987; 0.993) |
| Rural population (per 10%) | 1.124  (1.084; 1.166) | 1.134  (1.093; 1.176) | 0.784  (0.705; 0.872) | 1.121  (1.082; 1.162) | 1.141  (1.100; 1.183) | 1.147  (1.106; 1.190) | 0.760  (0.686; 0.842) | 1.142  (1.103; 1.183) |
| Fertility rate | 1.064  (1.034; 1.095) | 1.061  (1.031; 1.093) | 1.141  (1.047; 1.242) | 1.101  (1.071; 1.132) | 1.155  (1.122; 1.188) | 1.151  (1.120; 1.185) | 1.249  (1.149; 1.357) | 1.195  (1.163; 1.229) |
| Drinking water (per 10%) | 0.948  (0.926; 0.970) | 0.949  (0.927; 0.971) | 0.925  (0.815; 1.049) | 0.959  (0.940; 0.980) | 0.942  (0.921; 0.964) | 0.942  (0.921; 0.964) | 0.896  (0.793; 1.011) | 0.954  (0.935; 0.974) |
| Health expenditure (PPP per 1000) | 0.914  (0.900; 0.927) | 0.913  (0.900; 0.927) | 0.937  (0.919; 0.954) | 0.738  (0.708; 0.768) | 0.907  (0.894; 0.920) | 0.907  (0.894; 0.920) | 0.930  (0.914; 0.947) | 0.722  (0.694; 0.751) |
| Clean cooking (per 10%) | 0.918  (0.901; 0.935) | 0.915  (0.899; 0.932) | 0.529  (0.457; 0.613) | 0.946  (0.931; 0.962) | 0.919  (0.902; 0.936) | 0.917  (0.901; 0.934) | 0.575  (0.500; 0.662) | 0.953  (0.938; 0.969) |
| N (number of observations) | 1895 | 1895 | 584 | 1311 | 1895 | 1895 | 584 | 1311 |
| Hausman test (P-value) | p < 0.001 | p < 0.001 | p < 0.001 | p < 0.001 | p < 0.001 | p < 0.001 | p < 0.001 | p < 0.001 |

Note: We reported ratios (i.e. exponential values of effect estimates) from regression models with log-transformed neonatal and infant mortality outcomes. Hausman Test indicated for each model that fixed effect model is the preferred model.

Abbreviations: VAT= value-added tax; GDP= Gross domestic product; PPP= Purchasing power parity, AIC= Akaike information criterion; BIC= Bayesian information criterion
