# Supplementary material for: Cigarette taxation and neonatal and infant mortality: A longitudinal analysis of 159 countries
Source: PLOS Glob Public Health. 2022 Mar 16;2(3):e0000042. doi: 10.1371/journal.pgph.0000042 (PMC10021450; doi:10.1371/journal.pgph.0000042)
Supplement: S1 Text — (DOCX) [file pgph.0000042.s009.docx]

S1 Text. The list of countries included in the analysis

Afghanistan, Algeria, Angola, Antigua and Barb, Argentina, Armenia, Austria, Azerbaijan, Bahamas, Bahrain, Bangladesh, Barbados, Belarus, Belize, Benin, Bolivia, Botswana, Brunei Darussala, Bulgaria, Burkina Faso, Burundi, CĂ´te d'Ivoire, Cabo Verde, Cambodia, Cameroon, Central African, Chad, Chile, China, Colombia, Comoros, Congo, Congo DRC, Costa Rica, Croatia, Cyprus, Czech Republic, Denmark, Djibouti, Dominican Republ, Ecuador, Egypt, El Salvador, Equatorial Guine, Eritrea, Estonia, Eswatini, Ethiopia, Fiji, Finland, Gambia, Georgia, Germany, Ghana, Greece, Grenada, Guatemala, Guinea, Guinea-Bissau, Guyana, Honduras, Hungary, Iceland, India, Indonesia, Iran, Israel, Italy, Jamaica, Jordan, Kazakhstan, Kenya, Kiribati, Kuwait, Kyrgyzstan, Laos, Latvia, Lesotho, Liberia, Lithuania, Luxembourg, Madagascar, Malawi, Malaysia, Maldives, Mali, Malta, Marshall Islands, Mauritania, Mauritius, Mexico, Moldova, Mongolia, Morocco, Mozambique, Myanmar, Namibia, Nepal, Nicaragua, Niger, Nigeria, North Macedonia, Norway, Oman, Pakistan, Palau, Panama, Papua New Guinea, Paraguay, Peru, Philippines, Poland, Qatar, Romania, Russian Federati, Rwanda, Saint Lucia, Saint Vincent an, Samoa, San Marino, Sao Tome and Pri, Saudi Arabia, Senegal, Serbia, Seychelles, Sierra Leone, Singapore, Slovakia, Slovenia, Solomon Islands, South Africa, South Korea, Spain, Sri Lanka, Sudan, Suriname, Sweden, Switzerland, Tajikistan, Tanzania, Thailand, Timor-Leste, Togo, Tonga, Trinidad and Tob, Tunisia, Uganda, Ukraine, United Arab Emir, United Kingdom, United States, Uruguay, Uzbekistan, Vanuatu, Venezuela, Vietnam, Yemen, Zambia, Zimbabwe
