# Supplementary material for: Cigarette taxation and neonatal and infant mortality: A longitudinal analysis of 159 countries
Source: PLOS Glob Public Health. 2022 Mar 16;2(3):e0000042. doi: 10.1371/journal.pgph.0000042 (PMC10021450; doi:10.1371/journal.pgph.0000042)
Supplement: Attachment — Submitted filename: Answer to reviewers - fin.docx [file pgph.0000042.s010.docx]

30 September 2021

Dear Dr Eke,

**Re: Cigarette taxation and neonatal and infant mortality: a longitudinal analysis of 159 countries**

**Manuscript Reference Number: PGPH-D-21-00545**

Thank you for your invitation to revise our study and thank you also to the Reviewers for their supportive and constructive feedback, which we have carefully considered. We have now made a number of revisions to the manuscript in light of this feedback. Below, we provide comments on each point and describe the changes to the manuscript that we made accordingly. We first reproduce the editorial and Reviewers’ feedback in full (*italicised*) before detailing our responses. Please note that in our response, the page and line references refer to the manuscript version with tracked changes. Furthermore, we would like to ask you to communicate further decisions primarily to Anthony Laverty (a.laverty@imperial.ac.uk) who is now corresponding author.

Yours sincerely,

Anthony Laverty on behalf of the co-authors

1. ***Response to Reviewer 1:***
   1.1. The manuscript has tried to assess the impact of cigarette taxation against neonatal/infant mortality, of course there are many confounding factors. This information could serve as an evidence for the advocacy towards smoking ban and or smoking free legislations. It would have been better if they included smoking free legislations in the objectives of their work

Response: We thank the reviewer for this point. In this paper, we focused on cigarette taxation and smoke-free legislation was only a control variable. Specific analyses on the individual impacts of smoke free legislation would be a different paper and would very likely require different models and data. For example, a specific study on this issue would ideally consider whether laws cover all public places or not, as well as issues such as adherence and subnational legislation, which we do not consider here. We do agree with the reviewer that this would be informative and we do have plans to conduct similar analyses as part of future work.

1.2. In the materials and methods, construction of time-series models by infant race for cigarette tax and price with infant mortality would have been better to be included as the outcome.

Response: We agree with the reviewer that this would be an interesting topic. However, to our knowledge, infant mortality by ethnicity/race is not available globally which precludes us from conducting such analyses.

1.3. If possible, the authors are encouraged to think of the impact of educational attainments of the smokers (if possible), mean inflation-adjusted per-capita income of the countries, and country random effects; so that it is possible to appreciate the role of cigarette tax on the deaths

Response: We thank the reviewer for this thoughtful comment. While we have not been able to assess individual-level education of smokers, we did control for country level female education attainment and gross domestic product per capita (GDP in PPP) in our model. This means that our models control for changes in the composition of the population over time. With regards to country-level random effects, we did consider this in our analyses but random effects provided similar findings to our final fixed-effects models, which we mentioned in the previous version:

Line 155-159: “The Hausman specification test indicated that the random-effects models provide coefficients of similar magnitude to the fixed-effects models, with the exception of the model for the association between taxes and cigarette consumption in HICs in which a random-effects model was also reported.”

1.4. The authors are expected to explain confident they are to conclude the cause of death is exclusively because of cigarettes; OR the reduction of death is exclusively because of taxation of cigarette? Because there are several reasons to mention. So it is better to bring justification for the confounding factors

Response: Our model estimates the impact of cigarette taxes on infant deaths, with impacts caused by reduced smoking among pregnant women and reduced secondhand smoke exposure among infants. We conducted a number of sensitivity analyses including different potential confounders and these models performed similarly to our main analyses. A key strength of our study is that we applied fixed-effect models which automatically control for all time-invariant variables (i.e. those that do not change from one year to another such as climate). However, other variables that change over time (e.g. healthcare expenditure) were indeed concerns for our study. To ensure that all of these relevant confounders are controlled for, we selected them based on their demonstrated impact on the outcomes and exposure variables in existing literature (1)(2). We discussed in the limitation section possible confounders that could not be controlled for due to data limitations (line 276-290).

1.5. I didn’t see results for specific excise, ad valorem, Value Added Tax (VAT)/ import duties/ other taxes separately and which of the types of the taxes had greater impact on the deaths?

Response: Figure 1 and Table S2 provide the numeric results which are described in line 217-221. We revised the Statistical analyses section to make it clearer that types of taxes were not observed in separate models, but different variables in the same model:

Line 159-162: “Three main panel regression models were fitted for each outcome with: i) total cigarette tax as a continuous term, which was further stratified by income level (HICs vs. LMICs); ii) total tax as a categorical variable (described above); iii) each type of tax as separate continuous variables in the same model.”

1.6. Did u see any disparity towards cigarette tax and infant/neonatal death among countries??

Response: Although we analysed separately high-income and low- and middle-income countries, we did not look at differences within these groups. We now added to the discussion that future research should investigate this issue.

Line 212-216: “In LMICs, a 10 percentage-point increase in total tax was associated with a 2.6% (95% CI: 1.9% to 3.3%) decrease in neonatal mortality and a 2.0% (95% CI: 1.3% to 2.7%) decrease in infant mortality. Estimates in HICs were in the same direction, however, confidence intervals were wide and crossed zero. Of the estimated 231,220 averted infant deaths in 2018, 99.2% were in LMICs (Fig 2).”

Line 312-314: “Finally, future research should further investigate the differences between countries in improving child survival by raising taxes since this link depends on a complex range of market forces, including the influence of the tobacco industry.”

1.7. I couldn’t find any of the figures in this text

Response: Figures are attached separately in line with the requirement of PLOS Global Public Health. It should be available from the editorial manager homepage.

1.8. Any hint for which countries are included for this specific study?

Response: Thanks for the suggestion. We now added the list of countries to the Supplementary Material (S1 Appendix).

1.9. Confounding factors are there to be discussed

Response: We selected the possible confounders based on the previous literature. Further, we discuss possible confounders that could not be captured due to data limitations.

Line 138-139: “Covariates were selected based on their previously demonstrated impact on the outcomes and exposure variables (1)(2)(3).”

Line 277-284: “We were unable to account for a number of factors which might influence the link between taxes and child survival, such as the lagged prevalence of smoking, the extent of illicit trade (36), tax, prices, and availability of other harmful tobacco and non-tobacco products, sales of single cigarettes that are more affordable for low-income groups (37) or healthcare reforms targeting neonatal and infant mortality directly. Data on such measures and tactics are not systematically and comparably available for the wide range of countries studied here, meaning that our findings should only be applied to individual countries with caution.”

1.10. There are countries with smoking free legislations and how did you see this issue?

Response: We controlled for MPOWER measures which include smoke-free legislation also.

Line 139-144: “All models were adjusted for (…) MPOWER tobacco control measures (excluding ‘Raise taxes’, as we analysed detailed taxation data, and scores for ‘Monitoring of tobacco use’, as this measure is not expected to have an impact on the outcomes). MPOWER scores come from relevant WHO reports which give a score from zero to four on each domain and we modelled these continuously.”

We have also now added this sentence to the limitations section:

Line 284-285: “Also, while we adjusted models for country level MPOWER scores, these may not fully capture granular details of tobacco control measures at the local level”

1.11. My other concern is that whatever legislation and smoking free legislation you impose, people who are addicted for cigarettes will continue to smoke…

Response: Previous literature is clear that public health policies such as taxation of tobacco can encourage individual smokers to quit and it is this which has driven down smoking prevalences in many locations over the past 30 years. But we did attempt to assess this and ran a secondary analysis to examine associations between taxation changes and cigarette consumption per capita (line 222-231). These found that increases in taxation were linked to decreased cigarette consumption, which is in line with previous studies on the subject.

1.12. In the recommendation part, What if you recommend smoking bans?

Response: We agree that smoke-free legislation is often effective, however, this paper focused on cigarette taxes and as such, we limited our recommendations to this policy. Nevertheless, we added a reference to show that smoke-free legislation had previously demonstrated child health benefits in some selected countries:

Line 138-139: “Covariates were selected based on their previously demonstrated impact on the outcomes and exposure variables (10)(13)(31).”

2. **Response to Reviewer 2:**

Abstract:

2.1. Write the aim of the study after the introduction

Response: We added the aim of the study to the introduction part of the abstract.

Line 30-31: “We evaluated longitudinal associations of cigarette taxes with neonatal and infant mortality globally.”

2.2. Abstract includes conclusions and recommendations not a discussion

Response: We rephrased the discussion part so it links our finding to the previous literature:

Line 54-57: “In line with limited existing evidence conducted in HICs, we found that raising taxes on tobacco was associated with a reduction in neonatal and infant mortality globally.”

Introduction

2.3. line 67 the referernce is a systematic review saying the studies on taxation on child health is fewer. I think this should be revised

Response: We have amended this sentence to be clearer that we mean there are fewer studies of this issue for children than adults. The systematic review we cite shows that only five studies observed the relationship between taxes and different primary health outcomes which could not be meta-analyzed “because studies on tax increases and smoking cessation services had variable outcome reporting and overlapping study populations.” This is indeed fewer studies and less strong evidence than those investigating adult outcomes (e.g. smoking behaviour or adult health).

Line 76-77: “Studies examining the impact of cigarette taxation on child health outcomes are fewer than among adults*.*”

Methods:

2.4. The study design: it is not a quasi-epxerimental study. there is no intervention applied by the researchers. It is more likely a secondary data analysis

Response: We thank the reviewer for this interesting point but we are using the term quasi-experimental as per Reeves (2017), where it estimates “a causal relationship using exogenous variation in the exposure of interest which is not usually directly controlled the researcher.” However, we recognize that indeed this concept is ambiguous, therefore, we added a reference to this sentence for clarity.

Results:

2.5. 2- table 1 i think it could be a supplementary

Response: We would prefer to keep Table 1 in the text given that variables are defined only in this table (so that we keep the text concise). Nevertheless, we would be happy to revise this if required.

2.6. when saying 2.6% reduction or decreas, no need for the minus (-). It is already understandable by the word decrease

Response: We removed the minus signs throughout the article where they are not needed, as suggested.

2.7. all tables are supplementary, i think the researcher can choose one or two to put them in the results

Response: To keep our paper focused on the key variables, we would prefer to keep the tables in the supplementary material text. The coefficients for the key variables are displayed in the Figures (See Results section). The supplementary tables only add to the Figures the coefficients for the covariates. We would like to keep these coefficients in the Supplement to avoid someone causally interpret these because of the following reasons:

Line 306-310: “While our current study did include information about other MPOWER policies as covariates, these should not be interpreted causally since the choice of models and covariates was driven by the need to adjust for potential confounders associated with taxation and child survival outcomes specifically.”

3. ***Response to Editor:***

Please amend your detailed Financial Disclosure statement. This is published with the article, therefore should be completed in full sentences and contain the exact wording you wish to be published. If you did not receive any funding for this study, please simply state: “The authors received no specific funding for this work.”

Response: We provided the suggested Financial Disclosure statement (Line 326).

3.1. Please note that your Data Availability Statement is currently missing the repository name and a direct link to access each database. If your manuscript is accepted for publication, you will be asked to provide these details on a very short timeline. We therefore suggest that you provide this information now, though we will not hold up the peer review process if you are unable.

Response: We only used publicly available data for this analysis thus we believe that uploading it to a repository was not necessary. We added the link to the datasets.

Line 109-111: “Data were collected from the World Bank Database, World Health Organization, and International Cigarette Consumption Database (22)(23)(24).”

3.2. ”Please provide separate figure files in .tif or .eps format only and remove any figures embedded in your manuscript file. Please ensure that all files are under our size limit of 20MB. For more information about how to convert your figure files please see our guidelines: <https://journals.plos.org/globalpublichealth/s/figures>. Once you've converted your files to .tif or .eps, please also make sure that your figures meet our format requirements.

Response: We provided separate figures in the requested format.

3.3. We have noticed that you have uploaded supporting information but you have not included a list of legends. Please add a full list of legends for all supporting information files (including figures, table and data files) after the references list.

Response: We added the list of legends.

3.4. Please review your reference list to ensure that it is complete and correct. If you have cited papers that have been retracted, please include the rationale for doing so in the manuscript text, or remove these references and replace them with relevant current references. Any changes to the reference list should be mentioned in the rebuttal letter that accompanies your revised manuscript. If you need to cite a retracted article, indicate the article’s retracted status in the References list and also include a citation and full reference for the retraction notice.

Response: We reviewed our reference list.

**References**

1. Filippidis FT, Laverty AA, Hone T, Been JV, Millett C. Association of cigarette price differentials with infant mortality in 23 european union countries. JAMA Pediatr [Internet]. 2017 Sep 18;171(11):1100–6. Available from: <http://dx.doi.org/10.1001/jamapediatrics.2017.2536>
2. Radó MK, van Lenthe FJ, Sheikh A, Been J V. Investigating the effects of comprehensive smoke-free legislation on neonatal and infant mortality in Thailand using the synthetic control method. EClinicalMedicine. 2020;
3. Faber T, Kumar A, Mackenbach JP, Millett C, Basu S, Sheikh A, et al. Effect of tobacco control policies on perinatal and child health: a systematic review and meta-analysis. Lancet Public Heal [Internet]. 2017 Sep 20;2(9):e420–37. Available from: http://dx.doi.org/10.1016/S2468-2667(17)30144-5
